# Caveolin-1 is a risk factor for postsurgery metastasis in preclinical melanoma models

**DOI:** 10.1097/CMR.0000000000000046

**Published:** 2014-05-01

**Authors:** Lorena Lobos-Gonzalez, Lorena Aguilar-Guzmán, Jaime G. Fernandez, Nicolas Muñoz, Mehnaz Hossain, Simone Bieneck, Veronica Silva, Veronica Burzio, Elena V. Sviderskaya, Dorothy C. Bennett, Lisette Leyton, Andrew F.G. Quest

**Affiliations:** a Cellular Communication Laboratory, Center for Molecular Studies of the Cell (CEMC), Advanced Center for Chronic Diseases (ACCDiS), School of Medicine, Universidad de Chile; b Department of Surgery, Hospital Clinico Universidad de Chile; c Andes Biotechnologies, Fundación Ciencias y Vida; d School of Biological Sciences, Universidad Andres Bello, Santiago, Chile; e Division of Biomedical Sciences, St. George’s, University of London, London, UK; f Freie Universität Berlin – Institut für Pharmakologie, Berlin, Germany

**Keywords:** caveolin-1, immunodeficient B6Rag1−/−, promoter of metastasis, surgical resection, syngenic immunocompetent C57BL/6

## Abstract

Melanomas are highly lethal skin tumours that are frequently treated by surgical resection. However, the efficacy of such procedures is often limited by tumour recurrence and metastasis. Caveolin-1 (CAV1) has been attributed roles as a tumour suppressor, although in late-stage tumours, its presence is associated with enhanced metastasis. The expression of this protein in human melanoma development and particularly how the presence of CAV1 affects metastasis after surgery has not been defined. CAV1 expression in human melanocytes and melanomas increases with disease progression and is highest in metastatic melanomas. The effect of increased CAV1 expression can then be evaluated using B16F10 murine melanoma cells injected into syngenic immunocompetent C57BL/6 mice or human A375 melanoma cells injected into immunodeficient B6Rag1−/− mice. Augmented CAV1 expression suppresses tumour formation upon a subcutaneous injection, but enhances lung metastasis of cells injected into the tail vein in both models. A procedure was initially developed using B16F10 melanoma cells in C57BL/6 mice to mimic better the situation in patients undergoing surgery. Subcutaneous tumours of a defined size were removed surgically and local tumour recurrence and lung metastasis were evaluated after another 14 days. In this postsurgery setting, CAV1 presence in B16F10 melanomas favoured metastasis to the lung, although tumour suppression at the initial site was still evident. Similar results were obtained when evaluating A375 cells in B6Rag1−/− mice. These results implicate CAV1 expression in melanomas as a marker of poor prognosis for patients undergoing surgery as CAV1 expression promotes experimental lung metastasis in two different preclinical models.

## Introduction

Cancer is a leading cause of death worldwide, whereby the large majority of patients succumb as a consequence of metastasis to secondary sites rather than tumour growth at the initial site. Skin cancer is currently considered the third most common human malignancy and its incidence is increasing worldwide at an alarming rate because of environmental and behavioural changes. Within this group of cancers, melanomas are the most dangerous form and account for the majority of skin cancer-related deaths. As for other cancers, metastasis is considered the major threat to patient survival. Identification of relevant prognostic molecular markers in this context is therefore of great interest.

Caveolins are a family of proteins that are generally implicated in the formation of plasma membrane-associated structures called caveolae, in cholesterol transport and in the control of a large variety of cell signalling events. To date, three isoforms have been identified in mammals: caveolin-1 (CAV1), CAV2 and CAV3. In the context of cancer, CAV1 is the most studied isoform and a considerable amount of data now suggests that this protein plays a dual role in this complex process 1–4. For melanomas, relatively little is known about the role of CAV1. Some initial reports implicated the protein more as a tumour suppressor in melanoma lines. For instance, CAV1 was suggested to function as a tumour suppressor in melanoma cells by disrupting GD3-mediated signalling events 5. More recently, exosomes containing CAV1 were detected in the serum of melanoma patients and associated with a poor prognosis because of their immune suppressive effects 6. Alternatively, knocking down CAV1 expression in a melanoma line that has elevated levels reduced proliferation and tumourigenicity 7. Despite such evidence, another recent report indicates that CAV1 blocks metastasis of malignant melanomas in a murine model 8. Thus, the role of CAV1 in melanoma and particularly the possible consequences of CAV1 expression for patient survival postintervention remain unclear.

Here, we first evaluated the expression of CAV1 in human melanocytes and melanomas and observed a gradual increase in the expression of this protein with progression of disease. We then determined how the augmented presence of CAV1 introduced into human A375 or murine B16F10 melanoma cells promoted metastasis to different organs depending on the model. Given the importance of surgical procedures as an initial line of defence to eradicate melanomas, we used human A375 cells in immunodeficient B6Rag1−/− mice and mouse B16F10 cells in syngenic C57BL6 mice to evaluate how CAV1 affected surgery outcome. Following initial subcutaneous tumour growth to a defined size/volume in the two mouse models, the tumours were removed by standard surgical procedures applied in humans. After another defined period, animals were killed and tumour formation at the initial site as well as metastasis to the lung were evaluated. Using this model, we provide direct experimental evidence that CAV1 expression in melanomas before surgery favours metastasis and hence represents a negative prognostic marker for the outcome of surgical procedures.

## Methods

Polyclonal anti-CAV1 antibodies were from Transduction Laboratories (Lexington, Kentucky, USA). Antiactin antibodies were from R&D Systems (Minneapolis, Minnesota, USA) and Sigma (St Louis, Missouri, USA), respectively. Goat anti-rabbit immunoglobulin-G and goat anti-mouse immunoglobulin-G antibodies coupled to horseradish peroxidase were obtained from BioRad Laboratories (Hercules, California, USA) and Sigma, respectively. Isopropyl β-d-1-thiogalactopyranoside (IPTG) was from Sigma. The EZ-ECL chemiluminescent substrate was from Biological Industries (Kibbutz Beit Haemek, Israel). Hygromycin was from Calbiochem, La Jolla, California, USA. Cell media and antibiotics were from Gibco-BRL, Scotland, UK. Foetal bovine serum (FBS) was from Hyclone, Logan, Utah, USA. All other reagents used for western blots or cell culture were from Sigma or of the highest grade available.

### Western blotting

Protein extracts were separated by SDS-PAGE on 12% minigels (BioRad), loading 50 μg total protein. Blots were blocked with 5% milk in 0.1% Tween-PBS and probed with antiactin (1 : 5000) or anti-CAV1 (1 : 3000) antibodies. Bound antibodies were detected with horseradish peroxidase-conjugated secondary antibodies. CAV1 protein levels were quantified by scanning densitometry, standardized to actin levels in the same samples and comparing in each set with normal human melanocytes assigned a reference value of 1 (mean±SD, from three different experiments).

### Animals

Pathogen-free immunodeficient B6Rag1−/− (B6.129S7 Rag1<tm1Mom>/J) mice were from Jackson Laboratories (Bar Harbor, Maine, USA), whereas C57BL/6 mice were obtained from the ISP (Santiago, Chile). Animals were kept in the animal facility of the Andes Biotechnologies and Immunology Laboratory, Universidad de Chile, respectively. Mice between 8 and 12 weeks of age were used for experiments (approved by the local bioethics committee, CBA0271CMUCH).

### Cells and culture condition

Human melanoma lines were obtained from Dr Meenhard Herlyn, Wistar Institute, Pennsylvania, USA (WM lines), and from Dr I. Hart, G. Moore and P. Parsons, and were grown in RPMI1640 medium with 10% FBS, 2 mmol/l l-glutamine, 100 U/ml penicillin, 100 µg/ml streptomycin and 10% CO_2_, with the exception of WM1650, which were grown in the same medium with 200 nmol/l tetradecanoyl phorbol acetate and 100 pmol/l cholera toxin. Hermes 3A immortal human melanocytes (information concerning these cell lines is available at *http://www.sgul.ac.uk/depts/anatomy/pages/WTFGCB.htm*), and NOHM-4 normal neonatal human melanocytes, were obtained from Cascade Biologics, now MSD Biologics (Billingham, UK).

B16F10 (mock), B16F10 (cav-1), A375 (mock) and A375 (cav-1) cells induced or not with IPTG have been described 4,9. All cells were cultured in RPMI supplemented with 10% FBS, 2 mmol/l glutamine and antibiotics (100 U/ml penicillin and 100 µg/ml streptomycin) at 37°C, 5% CO_2_. The NhuM melanocyte cell line (ATCC Pcs-200-012) and the HEKn keratonocyte line (HEKn-APF Cascade Biologicals C-020-5C) were cultured in the Dermal Cell Basal Medium Kit (ATCC Pcs200-030; ATCC, Manassas, Virginia, USA) and KeratinocyteSFM (Gibco 10725018 suppl37000-015; Gibco, Carlsbad, California, USA), respectively.

### Immunohistochemistry

The tissue array used in this study, referred to as MEL961, includes 48 tissue sections of melanomas at different stages and also normal tissue (*http://www.pantomics.com/TissueArrays/Skin/Human/MEL961.aspx*). Tissue arrays were deparaffinated and hydrated following standard procedures. Antigenicity was recovered treating samples with Retrieval solution (S1699; DACO, Rune Linding, Denmark) at 100°C for 20 min. After washing, tissue arrays were blocked with goat serum 2% (diluted in PBS 1×) for 30 min and incubated with the primary antibody (1 : 100 in goat serum 2%) overnight. Samples were then washed 3× for 5 min with PBS 1×. First, antibodies were subsequently detected using the UltraVision LP Detection System-HRP Polymer & DAB Plus Chromogen (TL-015-HD; Thermo Scientific, West Palm Beach, Florida, USA) following the instructions provided by the manufacturer. Finally, samples were counterstained with haematoxylin, dehydrated and mounted in permanent medium Entellan (Merck, Darmstadt, Germany).

### Tumour growth assays

A375 (2 000 000 or 5 000 000) or B16F10 (300 000) cells were injected subcutaneously into the flanks of B6Rag1−/− or C57BL/6 mice, respectively. The appearance of tumours was monitored by palpitation (tumor volume=width^2^×length×*π*/6). In initial experiments using B16F10 cells, mice were injected simultaneously in the left and right flank with different cell lines [B16F10 (mock) and B16F10 (cav-1)] (Fig. 3). However, because of the considerable differences in tumour growth rates, mice were injected with only one cell line for the subsequent surgery experiments (Figs 4 and 5).

### Metastasis assays

Immunodeficient B6Rag1−/− mice injected intravenously with 5 000 000 A375 (mock) or A375 (cav-1) cells were killed day 21 after injection; the liver and lungs were fixed in Fekete’s solution. Metastasis was evaluated by analysing photographs. Because A375 are nonpigmented cells, tumour nodules apparent at the surface of liver and lung were determined (Fig. 2e and f). Animals injected intravenously with 200 000 B16F10 cells were killed day 21 after injection and lungs were fixed in Fekete’s solution. Black tissue, corresponding to lung metastases, was separated from the rest of the lung and weighed. Metastasis is expressed as black tissue mass/total lung mass (%). In these experiments, mice were evaluated according to established criteria 10–12.

### Surgery model

C57BL6 mice were injected subcutaneously with B16F10 (mock) or B16F10 (cav-1) (3×10^5^/100µl in NaCl 0.9%), (Figs 3 and 5) using two different procedures: (a) animals were injected with tumour cells on different days to allow operating on equal-sized tumours the same day. Mice were injected on day 0 with B16F10 (cav-1) and on day 4 with B16F10 (mock) cells (Fig. 5a). (b) All animals were injected the same day with the B16F10 (mock) and B16F10 (cav-1) cells and tumours were surgically removed as they reached 1500–1800 mm^3^. A comparison of tumour volumes for all experiments showed no significant differences between the animals injected with B16F10 (mock) and B16F10 (cav-1) cells (Fig. 4c). Tumours were removed using standard techniques applied in humans (complete and en-bloc resection). When tumour margins were visually compromised, surgical margins were widened so that no tumour mass was macroscopically visible. The open surgery wound was washed with saline solution and then sutured with silk 3.0 threads. Mice were treated with palliative medication. All the remaining mice were eventually killed on day 14 after surgery. Lung metastasis is expressed as black tissue mass/total lung mass (%, Fig. 6). In the immunodeficient surgical model, B6Rag1−/− mice were injected subcutaneously with 5 000 000 A375 (mock) or A375 (cav-1) cells and tumours were surgically removed when they reached 850–1100 mm^3^ (Fig. 6).

### Extumour cells

Developing tumours (Fig. 3) were surgically removed when they reached volumes of 1500–1800 mm^3^. Gelatinous material from the tumour interior was trypsinized for 15 min at 37°C, and then mechanically teased apart with tweezers and suspended in medium. The cells (mainly melanomas) were left to adhere to the plate for 6 h. Then, fresh culture was added. All cells were cultured in RPMI supplemented with 10% FBS, 2 mmol/l glutamine and antibiotics at 37°C, 5% CO_2_. Upon reaching 90% confluence, cells were trypsinized and replated again. Cells obtained after two passages in culture are referred to as B16F10 extumour cells.

### Statistical analysis

The results of Western blot experiments were compared statistically using the Kruskal–Wallis test. In-vivo experiments were compared using unpaired *t*-tests. A value of *P* less than 0.05 was considered significant.

## Results

### Increased CAV1 expression with melanoma malignancy

**Fig. 1:**
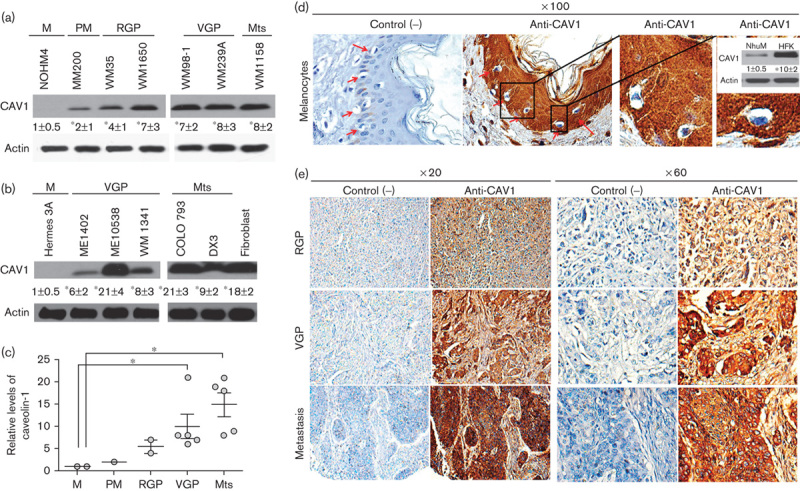
CAV1 levels in human melanocytes and melanoma cell lines. Human melanocytes and melanoma cell lines were grown in 100 mm plates (see the Methods section). At 70% confluence, cells were harvested, extracts were prepared and proteins were separated by SDS-PAGE in 12% minigels (50 µg total protein per lane), transferred to nitrocellulose and analysed by western blotting with anti-CAV1 and antiactin antibodies. CAV1 protein levels were quantified by densitometric analysis. Numerical data were normalized to actin and averaged from three independent experiments (mean±SD, *P<0.05). (a, b) Representative blots are shown for the following: Hermes 3A and NOHM-4 (normal human melanocytes, M); MM200 (primary melanoma, PM), WM35 and WM1650 (radial growth phase, RGP); ME1402, Me10538, WM1341, WM98-1 and WM239A (vertical growth phase, VGP) and WM1158, COLO793 and DX3 cells (metastatic, Mts). Human fibroblasts were included as a positive control for CAV1 expression. (c) Relative CAV1 expression levels are graphed for the different human melanoma preparations. Statistically significant differences are indicated (*P<0.05). (d) Immunodetection of CAV1 in human samples using tissue arrays (normal tissue sample A04; MEL961 Pantomics). Individual melanocytes within the keratinocyte layer are highlighted (red arrows). Note that CAV1 was essentially undetectable in normal melanocytes when compared with keratinocytes (100×). Western blot insert: NhuM cells (melanocyte cell line) and HEKn (keratinocyte cell line) were compared. Note that CAV1 levels were at least 10-fold higher in HEKn cells. (e) CAV1 expression was determined as described for different stages of melanoma development. In the order of increasing invasiveness, representative samples of RGP, VGP and higher metastatic state are shown (E06, F11, H12; respectively; MEL961 Pantomics). CAV1, caveolin-1.

In the normal skin, melanocytes are closely associated with basal keratinocytes. With the onset of melanocytic naevus formation, increased numbers of the morphologically atypical melanocytes are detected in the basal layer. In the radial growth phase (RGP), pigmented cells spread out essentially horizontally and can also lose contact with the keratinocytes. Then, in the vertical growth phase (VGP), the number of pigmented cells increases considerably and foci penetrate the dermis and may enter subcutaneous layers. Finally, metastatic cells (Mts) detach from the initial site and migrate to nearby or distant organs 13. Here, we compared by western blot analysis CAV1 levels in human melanocytes with those of primary malignant RGP, VGP and Mts cells and detected a highly significant increase in CAV1 expression with increasing progression of disease (Fig. 1a). This observation was corroborated in an analysis comparing additional VGP and Mts cell lines. In this case, fibroblasts were included as a positive control for CAV1 expression. For some VGP and Mts lines, CAV1 expression was as high as in the fibroblast controls (Fig. 1b). All numerical data shown in Fig. 1a and b were then compared graphically and highly significant increases compared with melanocyte expression levels (reference value 1) were obtained for VGP as well as Mts lines (Fig. 1c). Taken together, these results show that progression of melanoma development in humans correlates with increased CAV1 expression.

To further corroborate this conclusion, we also analysed sections of human tissue samples with melanocytes and melanomas at different stages using tissue arrays. As expected on the basis of the aforementioned observations, the intensity of CAV1 staining increased with progression of the disease. For melanocytes in normal tissue, low levels of CAV1 were detected. Specifically, this is apparent from the comparison of CAV1 staining in keratinocytes (high) versus melanocytes (low) in normal tissue samples (Fig. 1d). Western blot analysis indicated that CAV1 expression in keratinocytes was at least 10-fold higher than in melanocytes (Fig. 1d, western blot insert). As predicted on the basis of data shown in Fig. 1c, this analysis using tissue arrays of samples from human patients confirmed that CAV1 expression increased as disease progressed through RGP to VGP and finally fully blown metastasis.

### CAV1 overexpression in A375 human melanomas suppressed tumour formation and promoted metastasis in immunodeficient mice

**Fig. 2:**
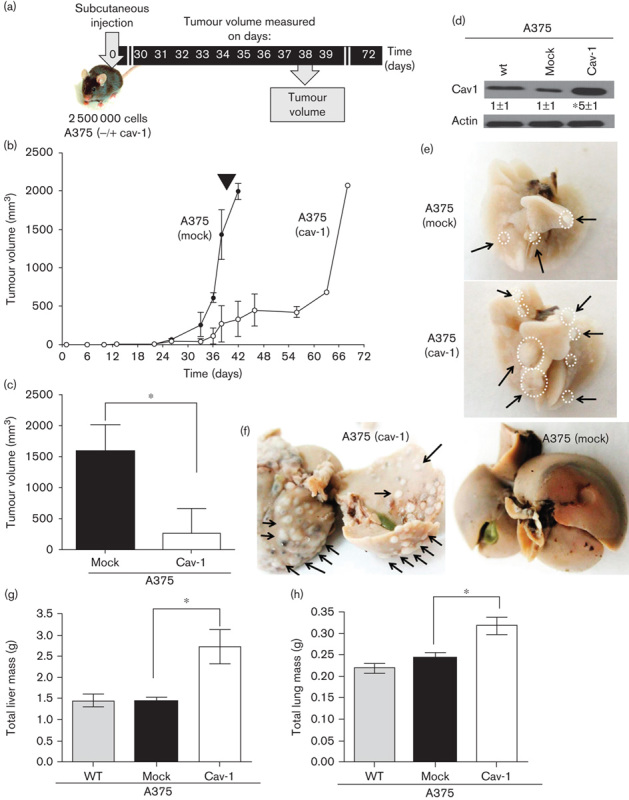
Tumour formation and lung metastasis of A375 (mock) and A375 (cav-1) cells. A375 (cav-1) and A375 (mock) cells were grown for 48 h in the presence of 1 mmol/l IPTG. Immunodeficient B6Rag1−/− mice were injected in the right flank with A375 (cav-1) or A375 (mock) cells (2.5×10^6^). (a) Schematic subcutaneous tumour formation. (b) Time course of tumour formation in B6Rag1−/− mice; black arrow corresponds to day 38. (c) Tumour volumes measured (day 38) for a total of five mice are shown. For CAV1-expressing cells and mock-transfected controls, tumour volumes were 268±232 and 1596±247 mm^3^, respectively (mean±SD). (d) Cell extracts from A375, A375 (mock) and A375 (cav-1) cells were analysed by western blotting with anti-CAV1 and antiactin antibodies. A representative western blot is shown. CAV1 protein levels observed in several experiments were quantified by scanning densitometry and normalized to actin (mean±SD, *n*=3; *P<0.05). A375 (mock) and A375 (cav-1) cells were injected intravenously (5×10^6^). Liver and lung tumour metastases were evaluated after 21 days. (e, f) Representative photographs of the liver (e) and lung (f) showing metastases by A375 (mock) and A375 (cav-1) cells (circles highlight tumours). (g, h) Quantification of total liver and lung mass evaluated 21 days after mice were injected with either A375 (mock) or A375 (cav-1) cells: liver 1.44±0.1 g compared with 2.73±0.4 g and lung 0.22±0.02 g compared with 0.32±0.02 g, respectively (*P<0.05). CAV1, caveolin-1; IPTG, isopropyl β-d-1-thiogalactopyranoside.

A375 cells were stably transfected with either an empty vector (pLacIOP) or vector encoding CAV1 (pLacIOPcav-1) to generate A375 (mock) and A375 (cav-1) cells, respectively. In A375 (cav-1) cells, CAV1 levels were significantly enhanced in the presence of IPTG used to induce expression (Fig. 2d). A time-course experiment showed that subcutaneous tumour formation was delayed for A375 (cav-1) cells (Fig. 2b). At day 38 after injection, tumour volumes were 268±232 and 1596±247 mm^3^ for A375 (cav-1) and A375 (mock), respectively (Fig. 2c). Of note, analysis of animal behaviour day 18 after injection, according to the criteria established by Morton^12^, yielded a score of 13, indicative of degenerated general health status and mobility for mice injected with A375 (cav-1) cells, whereas this was not the case for A375 (mock) cells (data not shown). Alternatively, when the same cells were injected intravenously, lung (Fig. 2e) and liver (Fig. 2f) metastasis was noticeably enhanced for A375 (cav-1) cells, whereas this was not the case for A375 (mock) cells. On average, for animals injected with A375 (cav-1) cells, the total liver mass increased at least 1.8-fold compared with the control animals (Fig. 2g), whereas increments in lung volume were less pronounced (1.5-fold; Fig. 2h).

Thus, although CAV1 shows characteristics typical of a tumour suppressor in subcutaneous tumour formation assays using human melanomas, these beneficial traits appear to be lost once cells reach the blood stream, as may occur unintentionally following surgery.

### Effect of CAV1 expression on B16F10 behaviour in tumour formation and metastasis assays

**Fig. 3:**
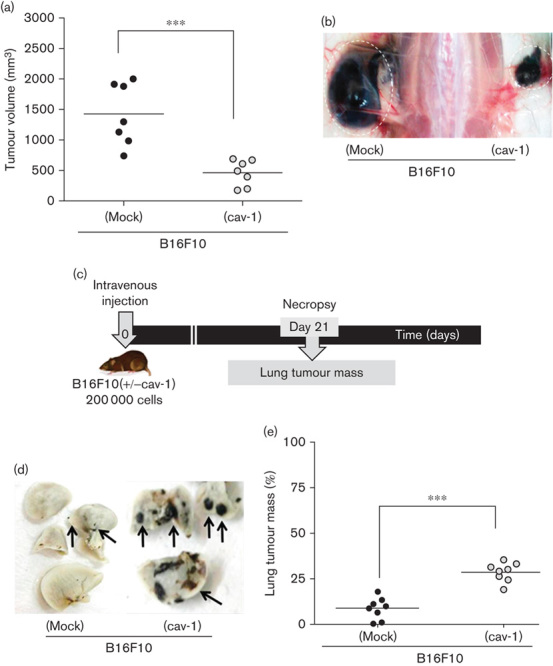
Tumour formation and lung metastasis of B16F10 (mock) and B16F10 (cav-1) cells. B16F10 (cav-1) and B16F10 (mock) cells were grown for 48 h in the presence of 1 mmol/l IPTG. C57BL/6 mice were injected simultaneously in the right flank with B16F10 (cav-1) and in the left flank with B16F10 (mock) cells (3×10^5^). (a) Tumour volumes measured (day 15) for a total of 14 mice. Means are shown. For CAV1-expressing cells and mock-transfected controls, tumour volumes were 460±81 and 1419±192 mm^3^, respectively (mean±SD). (b) Photograph of a necropsied mouse showing tumours formed in the left and right flank by B16F10 (mock) and B16F10 (cav-1) cells, respectively. (c) Schematic summarizing lung metastasis experiments. (d) Photographs showing lung metastasis by B16F10 (mock) and B16F10 (cav-1) cells. The black arrows indicate melanoma nodules. (e) C57BL/6 mice were injected with either 2×10^5^ B16F10 (cav-1) or B16F10 (mock) melanoma cells. Lung tumour mass was quantified after 21 days. Data from a total of 16 mice and the means are shown. Lung tumour mass for B16F10 (mock) and B16F10 (cav-1) cells was 9±2 and 28±2%, respectively (mean±SD). Statistically significant differences are indicated (***P<0.001). CAV1, caveolin-1; IPTG, isopropyl β-d-1-thiogalactopyranoside.

To corroborate these findings, B16F10 murine melanoma cells were stably transfected with either an empty vector (pLacIOP) or vector encoding CAV1 (pLacIOPcav-1) to generate B16F10 (mock) and B16F10 (cav-1) cells, respectively. The advantage of this model for the subsequent surgery experiments is that the tumour borders are better defined; metastasis occurs almost exclusively to the lung, facilitating quantification, and fully immunocompetent mice are used for the assays. In B16F10 (cav-1) cells, CAV1 expression was significantly enhanced both in the absence and in the presence of IPTG used to induce expression (Fig. 5). These cells were then evaluated in syngenic C57BL/6 mice for their tumour-forming ability (Fig. 3). Consistent with a role for CAV1 as a tumour suppressor, tumour formation of B16F10 (cav-1) cells was delayed compared with B16F10 (mock) cells at all time points (data not shown). Numerical analysis of the data at day 15 showed that volumes of tumours formed by B16F10 (cav-1) cells were significantly lower (Fig. 3a and b). We then evaluated the metastatic potential of these cells. Black lung tumour mass because of metastasis was recorded on day 21 after an intravenous injection (Fig. 3c). The black tumour tissue was visible and well defined, in the otherwise white parenchymal lung tissue (Fig. 3d), and metastatic mass was 8 and 28% of the total lung mass for B16F10 (mock) and B16F10 (cav-1) cells, respectively (Fig. 3e). Therefore, the presence of CAV1 in melanoma cells that were introduced directly into the blood stream elevated the metastatic potential of these cells. Importantly, spontaneous metastasis to the lung from subcutaneous tumours was not observed (data not shown).

### Effect of CAV1 presence on B16F10 behaviour in an immunocompetent postsurgery model

**Fig. 4:**
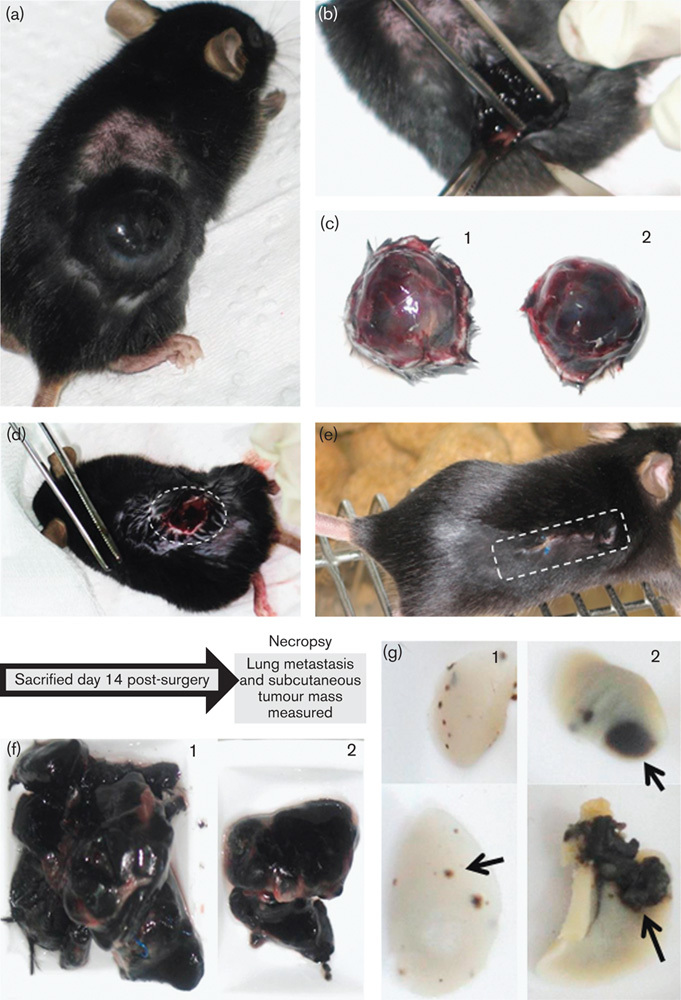
Melanoma surgery model. (a) C57BL6 mouse with a tumour of the bioethical permitted size (1500–1800 mm^3^). (b) Surgical removal of the tumour. (c) Surgically removed tumours of the same size derived from B16F10 (mock) (c1) and B16F10 (cav-1) cells (c2). (d) An animal immediately after surgery is shown with the open wound. (e) Operated animal, 2 days after surgery. (f) Following necropsy, 14 days after the initial surgery, recurrent tumours at the primary site were removed and volumes were measured. Tumours derived from B16F10 (mock) (f1) and B16F10 (cav-1) (f2) cells are shown. Note that tumours formed at the initial site after surgery by B16F10 (cav-1) cells were smaller than those obtained with B16F10 (mock) cells. (g) Lungs of mice killed 14 days after tumour surgery were analysed in animals initially injected subcutaneously with B16F10 (mock) (g1) or B16F10 (cav-1) (g2) cells. Note that lung metastasis was more prevalent for B16F10 (cav-1) cells.

We then sought to develop a mouse model to evaluate how CAV1 presence in subcutaneous melanoma tumours might impact on their potential to regenerate tumours at the initial site as well as to metastasize to the lung. Of the two available cell/animal models, we chose to continue studying B16F10 cells in C57BL/6 mice because tumour borders were better defined and the mice used are immunocompetent, as is the case for human patients. B16F10 melanoma cells expressing or not CAV1 were injected subcutaneously and tumours were allowed to grow to a defined size (Fig. 4a; 1500–1800 mm^3^). Then, the initial tumour was surgically removed (Fig. 4b and c), the wound was sutured and subsequent recovery was monitored (Fig. 4d and e). It should be noted that at the time of surgery, tumours formed by CAV1-expressing melanoma cells were of essentially the same size as those formed by B16F10 (mock) cells (compare Fig. 4c1 and c2).

On day 14 after surgery, animals were killed and tumour volumes at the initial site were determined. Consistent again with the notion that CAV1 functions as a tumour suppressor, tumours formed by B16F10 (cav-1) cells were smaller in comparison with B16F10 (mock) cells at the initial site of tumour growth before surgery (compare Fig. 4f1 and f2, respectively). However, analysis of tumour metastasis to the lung in the same mice showed an increase for CAV1-expressing cells in comparison with mock cells (Fig. 4g1 and g2, respectively).

**Fig. 5:**
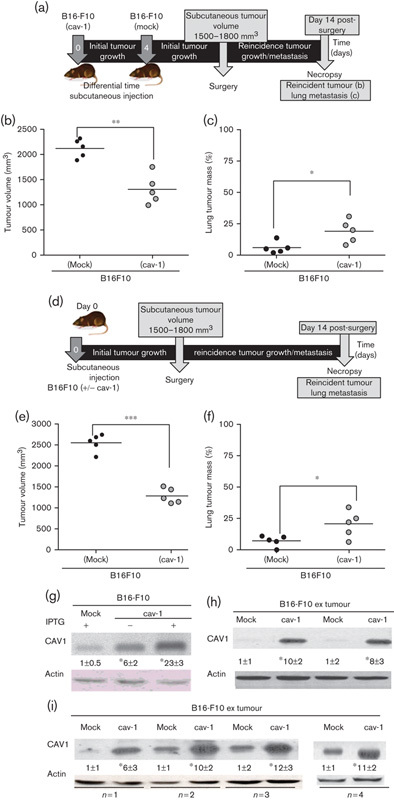
CAV1 suppressed tumour recurrence and increased lung metastasis 14 days after surgery. (a) Schematic of events in surgery experiments; mice were injected on different days. Tumours between 1500 and 1800 mm^3^ in size were surgically removed. Animals were monitored after surgery and reincidence of tumour growth as well as lung metastasis were evaluated after sacrificing the animals 14 days later. (b) Tumours formed after surgery by B16F10 (cav-1) cells were smaller than those derived from B16F10 (mock) cells: 1280±80 and 2543±94 mm^3^, respectively. (c) Lung metastasis was significantly elevated for B16F10 (cav-1) compared with B16F10 (mock) cells: 20±5 and 7±2%, respectively. (d) Schematic of events in surgery experiments using mice injected the same day with B16F10 cells. Tumours between 1500 and 1800 mm^3^ in size were surgically removed. Reincidence of tumour growth as well as lung metastasis were evaluated after killing the animals 14 days later. (e) Tumours formed after surgery by B16F10 (cav-1) cells were smaller than those derived from B16F10 (mock) cells: 1200±98 and 2543±94 mm^3^, respectively. (f) Lung metastasis was significantly elevated for B16F10 (cav-1) compared with B16F10 (mock) cells: 19±4 and 6±2%, respectively. Statistically significant differences are indicated (***P<0.001, **P<0.01, *P<0.05). B16F10 (mock) and B16F10 (cav-1) cells were grown 48 h in the absence (−) or presence (+) of 1 mmol/l IPTG. Cell extracts were separated by SDS-PAGE and analysed by western blotting with anti-CAV1 and antiactin antibodies. Results from a representative western blot are shown. CAV1 protein levels quantified by scanning densitometry and normalized to actin (mean±SD, *n*=3; *P<0.05) are shown before injection of cells into animals (g). Tumours generated by B16F10 (mock) and B16F10 (cav-1) cells grown to the same final volume (1500–1800 mm^3^) (Fig. 4c1, c2) or grown to different volumes and excised the same day (Fig. 2b and c) were analysed for CAV1 protein levels. (h, i) Cells extracted from tumours in Fig. 2b and c were cultured for two passages (B16F10 extumour cells) and protein extracts were prepared. Statistically significant differences compared with B16F10 (mock) cells are indicated (*P<0.05). CAV1, caveolin-1; IPTG, isopropyl β-d-1-thiogalactopyranoside.

Quantification of results from several experiments (Fig. 5a and d; time line of experiments) confirmed that tumours formed by CAV1-expressing B16F10 cells remained smaller at the primary site (Fig. 5b; **P<0.01 and Fig. 5e; ***P<0.001). However, lung metastasis was significantly elevated after surgery in those mice that had originally been inoculated subcutaneously with B16F10 (cav-1) cells (Fig. 5c; *P<0.05 and Fig. 5f; *P<0.05). Thus, in this animal model for melanoma surgery, we found that CAV1 expression in B16F10 cells generated less tumour mass at the primary site and yet enhanced metastasis to the lung. Of note, metastasis to other sites in the body, such as ganglia, was only detected in a few cases (data not shown).

### CAV1 expression in B16F10 extumour melanoma cells

We evaluated CAV1 expression levels in B16F10 (mock) and B16F10 (cav-1) cells before subcutaneous injection and after recovery from the primary tumours (Fig. 5g–i). As indicated previously, basal CAV1 levels were roughly six-fold higher in B16F10 (cav-1) than B16F10 (mock) cells and this difference increased by another 3–4 times in the presence of IPTG (Fig. 5g). When extumour cells were compared in the same way (Fig. 5h and i), significantly higher CAV1 levels were always detected in those cells isolated from B16F10 (cav-1) tumours. Note that although the basal levels of CAV1 were elevated in some cases, CAV1 levels were always higher in B16F10 (cav-1) than B16F10 (mock) extumour cells from the same mouse. These observations indicate that elevated CAV1 expression in melanomas is maintained throughout the subcutaneous tumour formation experiment.

### Effect of CAV1 presence on A375 behaviour in an immunodeficient postsurgery model

**Fig. 6:**
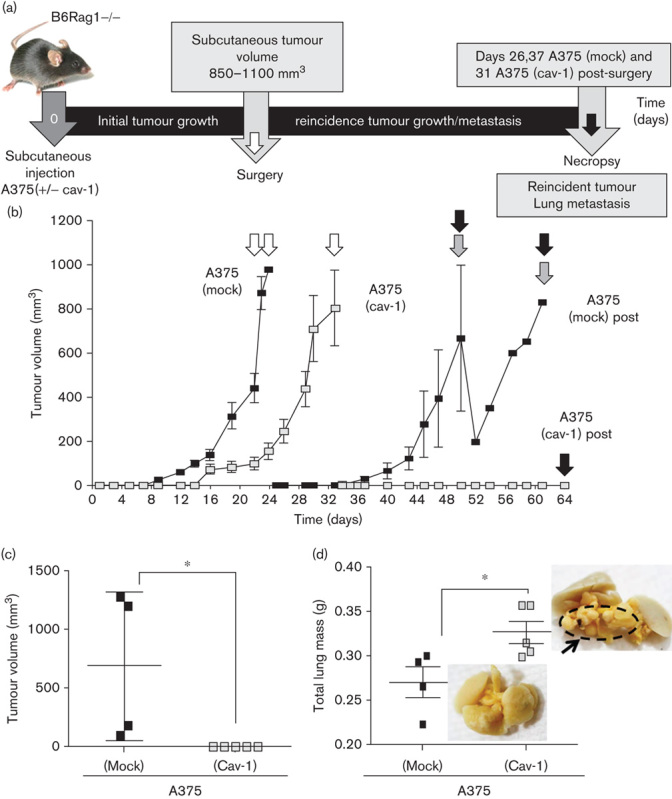
CAV1 suppressed tumour recurrence and increased lung metastasis of human melanomas after surgery in an mmunosuppressed mouse model. Immunodeficient B6Rag1−/− mice were injected into the right flank with either A375 (cav-1) or A375 (mock) cells (5×10^6^). (a) Schematic sequence of events in surgery experiments. Tumours between 800 and 1000 mm^3^ in size were surgically removed (white arrow). Animals were monitored after surgery and reincidence of tumour growth as well as lung metastasis were evaluated after killing the animals (black arrow in each case). (b, c) No tumours were detected after surgery for A375 (cav-1) cells. Alternatively, for A375 (mock) cells, mean tumour volumes of 697±310 mm^3^ were observed 50 days after surgery. Lung metastasis was significantly elevated after surgery for A375 (cav-1) compared with A375 (mock) cells. Quantification of total lung mass evaluated on the necropsy days (black arrow in b) (d) after mice were injected with either A375 (mock) or A375 (cav-1) cells: lung 0.275±0.05 g compared with 0.328±0.03 g, respectively (mean±SD), in the picture indicate the tumour mass in the case of A375 (cav-1) cells (*P<0.05). CAV1, caveolin-1.

Although for A375 (cav-1) cells local tumour recurrence after surgery was not detected, A375 (mock) cells develop tumours of 1000 mm^3^ volume or more between 50 and 61 days after surgery (Fig. 6b and c). In contrast, metastasis was noticeably enhanced for A375 (cav-1) cells (1.2-fold), whereas this was not the case for A375 (mock) cells (Fig. 6d).

## Discussion

The identification of molecular markers in cancer patients is becoming increasingly important to improve diagnosis as well as for the development of more refined treatments and prognosis following intervention. Here, we show first that CAV1 expression increased as human melanocytes progressed through the different stages to metastatic melanomas. Then, the consequences of elevating CAV1 expression to levels comparable with those detected in the human melanomas were evaluated in two animal models of experimental metastasis. In both cases, elevated CAV1 promoted metastasis. Finally, using one of these models, CAV1 was shown also to promote metastasis in a postmelanoma surgery setting. Together, these studies identified CAV1 as a protein whose expression increases with human melanoma progression and as a factor that favours lung metastasis in preclinical murine postsurgery settings.

After patients are operated (usually complete resection of the primary tumour), few clues are available, except for the Breslow index, to indicate how the disease will evolve in any given individual. This insight would be helpful because novel therapies are becoming available that hold promise in treatments following surgical resection of the initial melanoma tumour 14–16. However, there are essentially no reliable markers to indicate which patients will be cured by surgery alone, which require complementary treatment to avoid metastasis and which will develop metastasis no matter what treatment is used. In this respect, our results implicate CAV1 expression in tumour cells at the initial site as a negative prognostic marker for surgery outcome because of the enhanced metastatic potential of these cells. Whether it is important for melanoma cells to maintain CAV1 expression elevated once metastasis has occurred is not clear. The experiments using the mouse melanoma model suggest that this might be the case because CAV1 levels were elevated in B16F10 (cav-1) extumour cells (Fig. 5h and i).

To enhance CAV1 expression in B16F10 cells, the IPTG-inducible expression vector pLacIOP was used. In the absence of IPTG, CAV1 expression was six-fold elevated in B16F10 (cav-1) cells with respect to B16F10 (mock) cells and the addition of IPTG enhanced expression another three- to four-fold (Fig. 5g). Here, it should be noted that the range of increased expression obtained in this system correlated remarkably well with increments detected in metastatic melanomas compared with melanocytes (Fig. 1). For experiments shown (Figs 3 and 5b,c and e–g) cells were cultured according to standard protocols in the presence of IPTG for 48 h before injection into mice 17. Analysis of extumour cells showed that in the absence of IPTG, elevated CAV1 expression levels were maintained throughout the experiments (Fig. 5e and f). These observations are consistent with our previously reported findings analysing CAV1 expression in HT29 colon cancer cells after tumour formation 17.

The A375 human melanoma cells have already elevated CAV1 levels compared with non-Mts (data not shown). Stable transfection with pLacIOP (cav-1) led to a five-fold increase in CAV1 expression (Fig. 2d). Although more modest than the changes observed for B16F10 cells (Fig. 5d), this increment was sufficient to reduce tumour formation by these cells, and yet favours metastasis upon intravenous injection. Thus, also for human melanoma cells introduced directly into the blood stream, augmented expression of CAV1 expression favoured metastasis, in this case not only to the lung but also to the liver. Molecular traits in CAV1, required for this dramatic switch in function, remain to be determined.

Our results using mouse B16F10 and human A375 melanoma cells show that augmented CAV1 protein levels enhance the metastatic potential of these cells. Consistent with this interpretation, the expression of CAV1 was increased in human melanomas in tissues and cell lines compared with melanocytes from patients, as evidenced by western blotting (Fig. 1a–c) and immunohistochemical (Fig. 1d and e) analysis. In this context, another recent publication should be mentioned 18. According to this study, CAV1 may be transferred from tumour cells to stromal cells (through exosomes or direct cell–cell transfer of membranes). Thus, a possible alternative interpretation is that our observations reflect a system in which intrinsic CAV1 levels in tumour cells may be transferred to stromal cell populations and condition a niche for metastatic melanoma cells. In such a way, the metastatic potential of the melanoma cells would not necessarily be enhanced, but instead, their establishment (and detection) at distal sites may be improved. This is an intriguing possibility that we cannot formerly exclude and may also contribute towards our observations. However, the characterization *in vitro* of both B16F10 and A375 melanomas shows that CAV1 expression enhances migration and Rac1 activation 4,19 as well as invasion in a matrigel assay (data not shown). These findings are consistent with our interpretation of the current results that the intrinsic metastatic potential of melanoma cells is increased by the presence of CAV1 as reported here.

In patients, CAV1 presence in tumours often correlates with a poor prognosis 18–22. Our results analysing human melanocytes and different stages of melanoma progression suggest that CAV1 expression is linked to increased metastatic potential 7 and follows a pattern similar to that reported previously for prostate cancer 23. In normal prostate tissue, CAV1 has not been detected, but expression increases upon tumour formation in mouse models and human patients 24–27, and CAV1 presence promotes metastasis of prostate cancer cells through an autocrine/paracrine mechanism 23,28. Moreover, levels of exosomes carrying CAV1 were significantly elevated in patients compared with healthy controls 6. In addition, secreted CAV1 detected in serum from patients with prostate cancer is now being considered as a novel target for treatment. Indeed, injection of anti-CAV1 antibodies reduced experimental lung metastasis in a mouse model of prostate cancer 23. It is intriguing to speculate that CAV1 expression may not only follow a pattern similar to that described for prostate cancer but also promotes metastasis by similar mechanisms. However, more research is required to substantiate such possibilities.

Intravenous injection of tumour cells into the tail vein of animals is a frequently used approach to evaluate metastasis. Here, two models were used to evaluate how CAV1 expression affects melanoma behaviours *in vivo*. Although a common experimental approach, intravenous injection does not faithfully recapitulate all events associated with tumour metastasis. In human patients, surgical resection remains one of the most effective treatments for melanomas, and yet, success is very much determined by whether metastasis occurs or not. Thus, we reasoned that it would be most appropriate to evaluate additionally the consequences of CAV1 expression in melanoma in a clinically more relevant setting for metastasis, akin to the situation in human patients, where subcutaneous tumours are surgically removed. To this end, B16F10 tumours were allowed to grow to a defined size and then surgically excised. After another 14 days, mice were killed and both subcutaneous tumour formation at the original site and metastasis to the lung (and elsewhere) were quantified. Here, two points are worth noting. First, a qualified human surgeon (J.G.F.) conducted these procedures following the same criteria used in humans. Second, the C57BL6 mice used here had a fully functional immune system. Thus, the results obtained in this manner are likely to bear greater relevance to the situation in human patients undergoing surgery. Under these experimental circumstances, CAV1 presence was identified as a postsurgery risk factor because expression in B16F10 melanomas favoured metastasis to the lung after intervention.

The data presented here are in agreement with a report by Felicetti *et al*. 7 indicating that CAV1 expression is associated with increased metastatic potential in different human melanoma cell lines. However, a report by Trimmer and colleagues suggested that CAV1 expression is reduced in human metastatic melanoma cell lines and blocks metastasis of malignant melanomas. In particular, CAV1 overexpression in B16F10 cells was shown to increase proliferation but reduce lung metastasis 8. The reason for this difference, particularly also with respect to our results shown here, is not clear. However, in a previous study from our group, we validated the notion that CAV1 expression is upregulated in metastatic melanomas in a meta-analysis including hundreds of different lines, showing that CAV1 mRNA levels are increased in invasive compared with proliferative melanoma lines 4. Moreover, we corroborated our previous findings with B16F10 cells in two different ways. On the one hand, using protocols similar to those we previously described for B16F10 cells, we show here for human melanoma cells that CAV1 functions as a tumour suppressor upon subcutaneous injection of melanoma cells, but favours metastasis to the lung when injected intravenously. In addition, we developed a new experimental protocol similar to that used by surgeons in patients and found again that CAV1 expression in mouse and human melanomas favours metastatic dissemination after tumour surgery.

### Conclusion

In summary, enhanced CAV1 expression in human melanomas is linked here to augmented metastatic potential. Moreover, CAV1 is identified as a negative prognostic marker in melanoma patients undergoing surgery because the presence of the protein at levels comparable to those observed in the human melanoma samples is directly associated in our preclinical surgery models with increased lung metastasis.
